# Optimized vaccine candidate MVA-S(3P) fully protects against SARS-CoV-2 infection in hamsters

**DOI:** 10.3389/fimmu.2023.1163159

**Published:** 2023-10-18

**Authors:** Rana Abdelnabi, Patricia Pérez, David Astorgano, Guillermo Albericio, Winnie Kerstens, Hendrik Jan Thibaut, Lotte Coelmont, Birgit Weynand, Nuria Labiod, Rafael Delgado, Dolores Montenegro, Eugenia Puentes, Esteban Rodríguez, Johan Neyts, Kai Dallmeier, Mariano Esteban, Juan García-Arriaza

**Affiliations:** ^1^Department of Microbiology, Immunology and Transplantation, Rega Institute, Laboratory of Virology, Molecular Vaccinology and Vaccine Discovery, KU Leuven, Leuven, Belgium; ^2^Department of Molecular and Cellular Biology, Centro Nacional de Biotecnología (CNB), Consejo Superior de Investigaciones Científicas (CSIC), Madrid, Spain; ^3^Centro de Investigación Biomédica en Red de Enfermedades Infecciosas (CIBERINFEC), Madrid, Spain; ^4^Department of Microbiology, Immunology and Transplantation, Rega Institute, Laboratory of Virology and Chemotherapy, Translational Platform Virology and Chemotherapy, KU Leuven, Leuven, Belgium; ^5^Department of Imaging and Pathology, Translational Cell and Tissue Research, Division of Translational Cell and Tissue Research, KU Leuven, Leuven, Belgium; ^6^Instituto de Investigación Sanitaria Hospital 12 de Octubre (imas12), Madrid, Spain; ^7^Department of Microbiology, Hospital Universitario 12 de Octubre, Madrid, Spain; ^8^Department of Medicine, Medical School, Universidad Complutense de Madrid, Madrid, Spain; ^9^Biofabri, O Porriño, Pontevedra, Spain

**Keywords:** COVID-19, SARS-CoV-2, variants of concern, MVA-S(3P) vaccine candidate, prefusion-stabilized spike, hamsters, immunogenicity, efficacy

## Abstract

The development of novel optimized vaccines against coronavirus disease 2019 (COVID-19) that are capable of controlling the severe acute respiratory syndrome coronavirus 2 (SARS-CoV-2) pandemic and the appearance of different variants of concern (VoC) is needed to fully prevent the transmission of the virus. In the present study, we describe the enhanced immunogenicity and efficacy elicited in hamsters by a modified vaccinia virus Ankara (MVA) vector expressing a full-length prefusion-stabilized SARS-CoV-2 spike (S) protein [termed MVA–S(3P)]. Hamsters vaccinated with one or two doses of MVA-S(3P) developed high titers of S-binding IgG antibodies and neutralizing antibodies against the ancestral Wuhan SARS-CoV-2 virus and VoC beta, gamma, and delta, as well as against omicron, although with a somewhat lower neutralization activity. After SARS-CoV-2 challenge, vaccinated hamsters did not lose body weight as compared to matched placebo (MVA-WT) controls. Consistently, vaccinated hamsters exhibited significantly reduced viral RNA in the lungs and nasal washes, and no infectious virus was detected in the lungs in comparison to controls. Furthermore, almost no lung histopathology was detected in MVA-S(3P)-vaccinated hamsters, which also showed significantly reduced levels of proinflammatory cytokines in the lungs compared to unvaccinated hamsters. These results reinforce the use of MVA-S(3P) as a vaccine candidate against COVID-19 in clinical trials.

## Introduction

Since its appearance in December 2019, severe acute respiratory syndrome coronavirus 2 (SARS-CoV-2) had a devastating global impact on human health (1). SARS-CoV-2 infection results in coronavirus disease 2019 (COVID-19), which can progress to severe and life-threatening complications (1). Despite the rapid development of several SARS-CoV-2 vaccines, which helped to reduce hospitalizations and mortality associated with COVID-19, the number of viral infections continues to rise (2, 3). This loss of vaccine efficacy can be attributed to the rapid emergence of new variants of concern (VoC) with enhanced transmissibility and/or partial immune escape properties, limited access to vaccination, and vaccination reluctance in some regions (2). Moreover, the waning of vaccine-elicited immunity over time makes revaccination with booster doses, administered as frequently as every six months, necessary to maintain immunity (2, 3). Since the start of the pandemic, numerous SARS-CoV-2 vaccine candidates have been developed using different platforms (https://covid19.trackvaccines.org/vaccines/). However, to date, vaccines approved for global use belong to four main platforms, namely, inactivated virus, messenger RNA (mRNA), adenovirus vector–based vaccines, and protein-adjuvanted vaccines (2). Recently, adapted versions of some of these vaccines have been developed to target both the ancestral Wuhan strain of the SARS-CoV-2 virus and specific omicron subvariants (4).

The main target for COVID-19 vaccine design is the SARS-CoV-2 spike (S) glycoprotein, which is involved in virus entry and represents the primary target for neutralizing antibodies (5). The S glycoprotein can occur in two different conformations, namely, prefusion and postfusion, being the prefusion state of the S trimer the most immunogenic antigen to be included in efficient vaccine candidates (5). We have previously shown that a non-replicating modified vaccinia virus Ankara (MVA) poxvirus vector expressing a non-stabilized full-length SARS-CoV-2 S protein (termed MVA-CoV2-S or MVA-S) from the ancestral Wuhan strain is highly immunogenic and effective in different animal models, such as mice (6–12), hamsters (13), and rhesus macaques (14). In addition, we have also generated a novel, optimized recombinant MVA virus, named MVA-S(3P) [also termed MVA-CoV2-S(3P)], expressing a prefusion-stabilized full-length S protein from the ancestral Wuhan strain containing three amino acid changes for prolines in the S2 region and inactivation of the furin cleavage site (10, 11). Interestingly, administration of a single intramuscular or intranasal dose of MVA-S(3P) to C57BL/6 wild-type and K18-hACE2 transgenic mice induced higher titers of binding IgG and neutralizing antibodies against parental SARS-CoV-2 and several VoC, as well as S-specific T-cell responses, than MVA-S (10, 11). Furthermore, a single intramuscular or intranasal dose of MVA-S(3P) protected all vaccinated K18-hACE2 mice from morbidity and mortality caused by a SARS-CoV-2 challenge, being more effective than MVA-S (10, 11).

In the present study, we explore the immunogenicity and protective efficacy of the MVA-S(3P) vaccine candidate in the Syrian hamster infection model, as a necessary step forward for clinical trials with this promising vaccine candidate.

## Materials and methods

### Animals and ethics statement

Female Syrian golden hamsters (*Mesocricetus auratus*) aged 6 to 8 weeks were purchased from Janvier Laboratories (Le Genest-Saint-Isle, France) and housed in pairs in ventilated isolator cages (IsoCage N Biocontainment System, Tecniplast, Buguggiate, Italy) with *ad libitum* access to food and water, at 21°C, 55% humidity, and 12:12 dark/light cycles. Extra bedding material and wooden gnawing blocks were provided as cage enrichment. Animals were acclimated for 4 days prior to the start of the study. Housing conditions and experimental procedures were approved by the KU Leuven animal experimentation ethical committee (license P065-2020) and in accordance with institutional guidelines approved by the Federation of European Laboratory Animal Science Associations (FELASA).

### Cells

Vero E6 cells (ATCC CRL-1586) were maintained in Minimum Essential Medium (MEM; Gibco-Life Technologies) supplemented with 10% fetal bovine serum (FBS; Integro), 1% L-glutamine (Gibco-Life Technologies), and 1% sodium bicarbonate (Gibco-Life Technologies) (at KU Leuven) or in Dulbecco’s modified Eagle’s medium (DMEM) supplemented with 10 mM 4-(2-hydroxyethyl)-1-piperazineethanesulfonic acid (HEPES; Gibco-Life Technologies), 1X non-essential amino acids (Gibco-Life Technologies), penicillin (100 U/ml, Sigma-Aldrich), streptomycin (100 mg/ml, Sigma-Aldrich), and 10% heat-inactivated FBS (Gibco-Life Technologies) (at CNB-CSIC). Cell cultures were maintained at 37°C in a humidified incubator containing 5% CO_2_. Endpoint viral titrations in Vero E6 cells were performed with a medium containing 2% FBS instead of 10%.

### MVA-S(3P) vaccine

The vaccine candidate MVA-S(3P) [also termed MVA-CoV2-S(3P)] expresses a human codon-optimized full-length prefusion-stabilized SARS-CoV-2 S protein (strain B.1, Wuhan) that contains three mutations in the furin cleavage site (R682G, R683S, and R685S) to prevent cleavage of the S protein in S1 and S2 domains, and three additional mutations to proline in the S2 region that stabilize the S protein in a prefusion conformation (A942P, K986P, and V987P); its generation was previously described (10, 11). The MVA-S(3P) vaccine used in this study was manufactured according to current Good Manufacturing Practice by Biofabri (Spain). MVA-S(3P) virus was grown in cultured chicken cells (DF-1), harvested, clarified, and purified by tangential flow filtration, and then packed into vials and stored at -15°C to -30°C. MVA-WT virus is an attenuated poxvirus strain, obtained from the Chorioallantois vaccinia virus Ankara strain after 586 serial passages in chicken embryo fibroblast (CEF) cells (15), and was grown in DF-1 cells and purified by centrifugation through two 36% (wt/vol) sucrose cushions in 10 mM Tris-HCl (pH 9). MVA virus titers were determined by immunostaining and reported as plaque-forming units (PFU) per ml, as previously described (16).

### SARS-CoV-2 virus

SARS-CoV-2 strain used in the *in vivo* study (BetaCov/Belgium/GHB-03021/2020, EPI_ISL_407976|2020-02-03) derived from the prototypic strain B.1 (Wuhan), and has been previously described (17). It was recovered from a nasopharyngeal swab taken from an RT-qPCR-confirmed asymptomatic patient who returned from Wuhan, China, in early February 2020. A close relationship to the prototype Wuhan-Hu-1 2019-nCoV (GenBank accession number MN908947.3) strain was confirmed by phylogenetic analysis.

The SARS-CoV-2 MAD6 isolate, used in the live neutralization experiments, is similar to the B.1 strain but contains the D614G mutation in the S protein; it has been previously described (7, 18).

SARS-CoV-2 virus stocks were grown in Vero E6 cells for two (MAD6) or three (B.1) passages. Virus stocks were free from mycoplasma (PlasmoTest, InvivoGen), and deep sequencing on a MiSeq platform (Illumina) confirmed that the stocks did not contain other adventitious agents. Infectious virus titers of viral stocks were determined by endpoint dilution in Vero E6 cells by the Reed and Muench method (19) and expressed as median tissue culture infectious dose (TCID_50_). All work related to live viruses was carried out at the A3 and BSL3+ high-containment facilities of the KU Leuven Rega Institute (3CAPS) in Belgium under the licenses AMV 30112018 SBB 219 2018 0892 and AMV 23102017 SBB 219 20170589, or at the BSL-3 facilities of the CNB-CSIC (Spain), according to the respective institutional guidelines.

### SARS-CoV-2 efficacy study schedule in hamsters

The SARS-CoV-2 hamster infection model has been previously described (13, 17, 20). The study comprised three groups of 10 hamsters. On day 0, group 1 (control group) received 1 × 10^8^ PFU/hamster of MVA-WT (as matched placebo), group 2 received PBS, and group 3 received 1 × 10^8^ PFU/hamster of MVA-S(3P). On day 28, group 1 received 1 × 10^8^ PFU/hamster of MVA-WT, and groups 2 and 3 received 1 × 10^8^ PFU/hamster of MVA-S(3P). All immunizations were performed intramuscularly in a total volume of 500 µl (250 µl in each leg). At days 0 (baseline, prior to the first immunization), 28 (prior to the second immunization), 49 (3 weeks after the second immunization), and 56 (prior to SARS-CoV-2 infection), blood samples (~500 µl) were collected from all animals under isoflurane anesthesia via the jugular vein. On day 56, all animals were infected intranasally with 2 × 10^5^ TCID_50_ of SARS-CoV-2 (B.1; BetaCov/Belgium/GHB-03021/2020, EPI_ISL_407976|2020-02-03) in 50 µl (approximately 25 µl/nare) culture medium (MEM, 2% FBS) under isoflurane anesthesia. After the SARS-CoV-2 challenge, hamsters were weighed daily and observed for mobility, self-maintenance, behavior, and humane endpoint (hind limb paralysis, hunchback, and sour eyes). On day 60 (4 days after SARS-CoV-2 infection), all hamsters were sacrificed by intraperitoneal injection of 500 µl of Dolethal (200 mg/ml sodium pentobarbital, Vétoquinol SA, Aartselaar, Belgium). Blood (~1 ml), nasal washes, and lungs were collected at the endpoint. Viral RNA in the lungs and nasal washes was quantified by RT-qPCR. Infectious virus yields in the lungs were quantified by endpoint virus titration. Additionally, the left lungs were collected in formaldehyde for histopathological analysis. After collection, the blood was centrifuged for serum preparation (10,000 g, 10 min, room temperature). The supernatant (serum) was collected in Eppendorf tubes and heat-inactivated at 56°C for 30 min, and the serum samples were stored at -80°C until use.

### Enzyme-linked immunosorbent assay

Individual serum samples obtained from hamsters at days 0, 28, 49, 56, and 60 were tested for the presence of binding IgG antibodies against SARS-CoV-2 S protein by ELISA, as previously described (7). The S protein (amino acid residues 1 to 1208) used to coat 96-well tissue culture plates was derived from the Wuhan-Hu-1 strain (GenBank accession number MN908947.3), but the furin-recognition motif Arg-Arg-Ala-Arg was replaced by the Gly-Ser-Ala-Ser sequence and also contained the Ala942Pro, Lys986Pro, and Val987Pro substitutions in the S2 portion to stabilize the S protein in a prefusion conformation. The use of a prefusion S protein in ELISA assays allows the measurement of vaccine-induced antibodies directed to functionally relevant epitopes on the S1 subunit. Total binding endpoint IgG titers were calculated as the reciprocal value of the last serum dilution giving an absorbance value at 450 nm at least three times greater than the absorbance of serum from day 0 (pre-immune serum).

### Live SARS-CoV-2 neutralization

Live-virus SARS-CoV-2 neutralizing antibodies were measured on day 56 (just before the SARS-CoV-2 challenge) using a microneutralization test (MNT) assay in a BSL-3 laboratory, as previously described (8, 10, 11). Serially, two-fold diluted serum samples in DMEM-2% FBS medium were incubated at a 1:1 ratio with 100 TCID_50_ of the SARS-CoV-2 MAD6 isolate in 96-well tissue culture plates for 1 h at 37°C. Then, mixtures of serum samples and SARS-CoV-2 virus were added in duplicate to Vero-E6 cell monolayers seeded in 96-well plates at 30,000 cells/well, and the plates were incubated at 37°C, in a 5% CO_2_ incubator for 3 days. Cells were then fixed with 10% formaldehyde for 1 h and stained with crystal violet. When the plates dried, the crystal violet was diluted in H_2_O-10% sodium dodecyl sulfate (SDS), and the optical density was measured in a luminometer at 570 nm. The neutralizing titer 50 (NT_50_) was calculated as the reciprocal dilution that results in 50% inhibition of cell death following a previously described methodology (21).

### Neutralization of SARS-CoV-2 variants of concern by pseudotyped virus serum neutralization test

In addition, neutralizing antibody titers against parental SARS-CoV-2 (isolate containing the D614G mutation) and several VoC (beta, gamma, delta, and omicron) were quantified on day 56 (just before SARS-CoV-2 challenge) using an in-house-developed serum neutralization test (SNT) with green fluorescent protein (GFP)-expressing vesicular stomatitis virus (VSV) pseudotypes carrying the SARS-CoV-2 S, as previously described (20). SARS-CoV-2 VSV pseudotypes were generated as follows: based on the plasmid backbone, BHK-21J cells (D614G and B.1.1.529 omicron, cloned in pCAGGS) or HEK-293T cells (B.1.351 beta, P.1 gamma, and B.1.167.2 delta, obtained from Invivogen Cat. No. plv-spike-v3, plv-spike-v5, and plv-spike-v8, respectively) were transfected with the respective S-protein expression plasmid. One day after transfection, cells were infected with VSVΔG virus expressing the GFP reporter for 2 h (22). The medium was exchanged with a medium containing anti-VSV-G antibody (I1, mouse hybridoma supernatant from ATCC CRL-2700) to neutralize any residual VSV-G virus input. After 26 h of incubation at 32°C, the supernatant containing SARS-CoV-2 VSV pseudotypes was collected.

Next, to quantify SARS-CoV-2 neutralizing antibodies, serial dilutions of serum samples were incubated for 1 h at 37°C with an equal volume of SARS-CoV-2 pseudotyped VSV particles and added in triplicate to Vero-E6 cell monolayers seeded in 96-well plates (10,000 cells/well). Then, the plates were incubated for 19 h, at 37°C, in a 5% CO_2_ incubator. The percentage of cells expressing GFP was quantified on a Cell Insight CX5/7 High Content Screening platform (Thermo Fisher Scientific) with Thermo Fisher Scientific HCS Studio (v.6.6.0) software. Values for the serial serum dilutions were normalized against a serum-free virus control (=100%), and SNT_50_ was calculated by fitting this dilution in GraphPad with non-linear regression (variable slope, four parameters, and top (100), bottom (0) constraints).

### RNA extraction and quantification of SARS-CoV-2 RNA and pro-inflammatory cytokines by RT-qPCR

Hamster lung tissues were harvested post-sacrifice on day 4 post-infection and homogenized using bead disruption (Precellys) in 350 µl TRK buffer (E.Z.N.A.® Viral RNA Kit, Omega Bio-Tek) and centrifuged (10,000 rpm for 5 min at 4°C) to precipitate cell debris and obtain clear supernatant. Then, RNA was extracted according to the manufacturer’s instructions. For nasal washes, 150 µl samples were used for RNA extractions using the NucleoSpin RNA Virus kit (Macherey-Nagel) according to the manufacturer’s protocol. Of 50 μl of eluate, 4 μl was used as a template in the RT-qPCR reactions. RT-qPCR was performed on a LightCycler96 platform (Roche) using the iTaq Universal Probes One-Step RT-qPCR kit (BioRad) with primers and probes targeting the SARS-CoV-2 nucleocapsid (N) gene region to analyze SARS-CoV-2 subgenomic (sgm) RNA, as previously described (17). Standards of SARS-CoV-2 cDNA (IDT) were used to express viral genome copies per mg of lung tissue or per ml of nasal wash.

The expression levels of the cytokines IL-6, MX-2, IP-10, and IFN-γ were analyzed in lung samples by RT-qPCR with primers and probes previously described (17). Expression levels of selected cytokines were normalized to β-actin expression and the relative fold change was calculated using the 2^-ΔΔCt^ method.

### Infectious virus titration

Lung tissues were homogenized by bead disruption (Precellys) in 350 µl MEM and centrifuged (10,000 rpm for 5 min at 4°C) to pellet cell debris and obtain the supernatant. To quantify infectious SARS-CoV-2 particles, serial dilutions of centrifuged, homogenized lung tissue supernatant were incubated for 3 days in confluent Vero-E6 cell monolayers seeded in 96-well plates. Infectious viral titers were calculated by the Reed and Muench method (19) and expressed as TCID_50_ per mg of lung tissue.

### Lung histopathology

For histological examination, the lungs were fixed overnight in 4% formaldehyde and then embedded in paraffin. Tissue sections (5 μm) were stained with hematoxylin and eosin and scored blindly by an expert pathologist for signs of lung damage, as previously described (23). Histopathologic parameters used to assess the SARS-CoV-2-induced lung pathology cumulative score were as follows: congestion, intra-alveolar hemorrhage, apoptotic bodies in the bronchial wall, necrotizing bronchiolitis, perivascular edema, bronchopneumonia, perivascular inflammation, peribronchial inflammation, and vasculitis.

### Statistical analysis

All graphs, calculations, and statistical analyses were performed using GraphPad Prism software version 9.4.1 (GraphPad Software). An unpaired non-parametric Mann-Whitney test of transformed data was used for the statistical analysis of IgG titers, and an unpaired non-parametric Kruskal-Wallis test of transformed data was used for the live virus and SARS-CoV-2 pseudotyped VSVs NT_50_ neutralizing titers. An ordinary one-way ANOVA followed by Tukey’s multiple comparison tests was employed for the statistical evaluation of percentages of body weight change, and ordinary one-way ANOVA of transformed data followed by Tukey’s multiple comparison tests for SARS-CoV-2 and cytokine mRNA levels and SARS-CoV-2 virus yields. For the statistical analysis of cumulative lung scores, an unpaired non-parametric Kruskal-Wallis test was performed. Statistical significance is indicated as follows: *p < 0.033; **p < 0.002; ***p < 0.0002; ****p<0.0001.

## Results

### MVA-S(3P) vaccine candidate induces high titers of anti-SARS-CoV-2 antibodies in vaccinated hamsters

**Figure 1:**
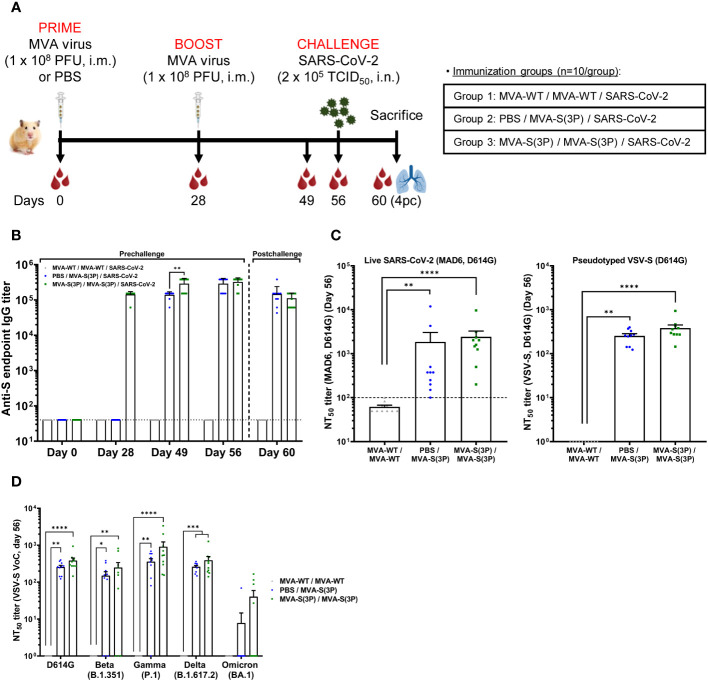
MVA-S(3P) immunization and SARS-CoV-2 challenge schedule in hamsters and analysis of SARS-CoV-2-specific humoral immune responses elicited by MVA-S(3P) vaccination. **(A)** Experiment overview. Syrian hamsters (n = 10 per group) were immunized intramuscularly (i.m.) with two doses of 1 × 10^8^ PFU of MVA-S(3P) or MVA-WT on days 0 and 28 or one dose of 1 × 10^8^ PFU of MVA-S(3P) on day 28 and challenged intranasally (i.n.) with 2 × 10^5^ TCID_50_ of SARS-CoV-2 B.1 strain on day 56, as indicated. Blood was collected on days 0, 28, 49, and 56. On day 60 (day 4 post-challenge; 4pc), all animals per group were sacrificed and blood, lungs, and nasal washes were collected for immunological, virological, and histological analysis. **(B)** Titers of anti-S binding IgG antibodies determined by ELISA in serum collected on days 0, 28, 49, 56, and 60. Mean values and SEM are plotted. The dashed line represents the limit of detection. Statistical significance between groups was calculated using an unpaired non-parametric Mann-Whitney test of transformed data (**p < 0.002). **(C)** SARS-CoV-2 neutralizing antibody titers against Wuhan isolate D614G were determined by a live microneutralization assay (left panel) or by S-pseudotyped VSV particles (right panel). NT_50_ (50% neutralization) titers were assessed in serum collected on day 56. Mean NT_50_ values ± SEM are depicted. The dotted line indicates the limit of detection. Statistical significance between groups was calculated using an unpaired non-parametric Kruskal-Wallis test of transformed data (**p < 0.002, ****p<0.0001). **(D)** SARS-CoV-2 neutralizing antibody titers against SARS-CoV-2 VoC beta, gamma, delta, and omicron were determined by an S-pseudotyped VSV particle assay. NT_50_ titers were assessed in serum collected on day 56. Mean NT_50_ values ± SEM are depicted. Statistical significance between groups was calculated using an unpaired non-parametric Kruskal-Wallis test of transformed data (*p < 0.033; **p < 0.002; ***p < 0.0002; ****p<0.0001).

We previously described that our MVA-S(3P) vaccine candidate, expressing a prefusion-stabilized SARS-CoV-2 S protein, was highly immunogenic and protected against SARS-CoV-2 morbidity and mortality in SARS-CoV-2-susceptible transgenic K18-hACE2 mice (10, 11), either through intramuscular (11) or intranasal (10) immunizations. Here, to further evaluate the immunogenicity and efficacy of the MVA-S(3P) vaccine in a second relevant animal model, a requirement for entry into phase I human clinical trials, groups of female Syrian hamsters (n=10 per group) were vaccinated with one or two doses of MVA-S(3P) and subsequently challenged with SARS-CoV-2 (**Figure 1A**). A group of hamsters (group 3) received a first (prime) dose of 1 × 10^8^ PFU of MVA-S(3P) intramuscularly on day 0, followed by a second (booster) dose of 1 × 10^8^ PFU of MVA-S(3P) on day 28 [MVA-S(3P)/MVA-S(3P)], whereas another group (group 2) only received a single dose of 1 × 10^8^ PFU of MVA-S(3P) on day 28 [PBS/MVA-S(3P)]. Primed and boosted hamsters inoculated with similar doses of 1 × 10^8^ PFU of MVA-WT on days 0 and 28 served as the matched placebo control group (group 1) (MVA-WT/MVA-WT) (**Figure 1A**). Then, four weeks after the last vaccine dose (day 56), all hamsters were challenged intranasally with 2 × 10^5^ TCID_50_ of SARS-CoV-2 and sacrificed on day 4 post-infection to collect different samples (serum, lungs, and nasal washes) that were used to evaluate the immunogenicity and efficacy of the MVA-S(3P) vaccine (**Figure 1A**). Immunization with one or two intramuscular doses of MVA-S(3P) was well tolerated in all hamsters with no signs of adverse effects, local reactogenicity, or changes in behavior during the vaccination period.

To measure the capacity of MVA-S(3P) to induce SARS-CoV-2-specific binding and neutralizing antibodies, serum samples were collected on day 0 (baseline, prior to first immunization in group 3), day 28 (prior to first immunization in group 2 or second immunization in group 3; 4 weeks after the first immunization in group 3), day 49 (3 weeks after the first immunization in group 2; 3 weeks after the second immunization in group 3), day 56 (prior SARS-CoV-2 infection; 4 weeks after the first immunization in group 2; 4 weeks after the second immunization in group 3), and day 60 (4 days after SARS-CoV-2 infection). First, we measured anti-S binding IgG antibody levels at all time points (days 0, 28, 49, 56, and 60) by ELISA. These data show that vaccination with one or two doses of MVA-S(3P) induced equally high IgG titers against the S protein (**Figure 1B**), with induction of high binding anti-S IgG antibody titers at 3-4 weeks after the first MVA-S(3P) dose, further enhanced at 3-4 weeks after the second (booster) dose, and then maintained after SARS-CoV-2 challenge (**Figure 1B**).

Next, we measured SARS-CoV-2 neutralizing antibody levels at day 56 (just prior SARS-CoV-2 challenge; 4 weeks after the first or second MVA-S(3P) dose) by using either a live microneutralization assay or a neutralization assay using S-pseudotyped VSV particles in both cases against SARS-CoV-2 virus or VSV-S particles containing the D614G mutation in the S protein. The results show that all vaccinated animals (10/10 in each vaccinated group) induced SARS-CoV-2 neutralizing antibodies with MVA-S(3P) double vaccination, eliciting higher neutralizing antibody titers against SARS-CoV-2 than the single immunization, but with no statistical significant differences between both vaccinated groups (geometric mean titer (GMT) +/- SEM: 2419,2 +/- 847,3 in the two-dose regimen and 1853,6 +/- 1187,2 in the single-dose regimen, using the live microneutralization assay; 382,6 +/- 68,1 in the two-dose regimen and 254,1 +/- 30,3 in the single-dose regimen, using S-pseudotyped VSV particles) (**Figure 1C**), and no neutralizing activity detected in the MVA-WT control group.

Finally, we analyzed the titers of neutralizing antibodies against several SARS-CoV-2 VoC on day 56 by using S-pseudotyped VSV particles expressing S proteins from SARS-CoV-2 VoC beta (B.1.351), gamma (P.1), delta (B.1.167.2), and omicron (B.1.1.529; BA.1) and compared with those induced by the SARS-CoV-2 B.1 strain (D614G). These data show that two doses of MVA-S(3P) induced higher titers of SARS-CoV-2 neutralizing antibodies than the one-dose schedule against the prototypic B.1 strain containing the D614G mutation (382,6 +/- 68,1 versus 254,1 +/- 30,3), and also against VoC beta (B.1.351; 246,7 +/- 91,9 versus 149 +/- 41,9), gamma (P.1; 905,9 +/- 322,4 versus 357,5 +/- 73,8), and delta (B.1.167.2; 389,9 +/- 104,8 versus 259,7 +/- 22,5), but with no statistical significant differences between both vaccinated groups (**Figure 1D**). The omicron BA.1 variant could be also neutralized (40,2 +/- 19,1 versus 7,8 +/- 6,8), but with a lower seroconversion rate and with 9,5-32,5-fold lower titers in comparison with B.1 in the two-dose and one-dose regimens, respectively, but again with no statistically significant differences between both vaccinated groups (**Figure 1D**).

### MVA-S(3P) vaccine candidate protects hamsters from SARS-CoV-2 challenge

**Figure 2:**
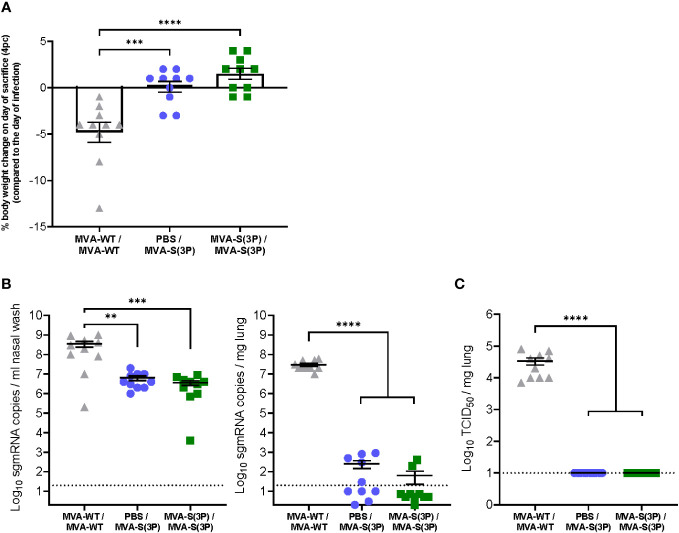
Hamster body weight change and SARS-CoV-2 virus replication in the lungs and nasal washes. **(A)** Percentage of hamster body weight change on the day of sacrifice (day 60, day 4 post-challenge) normalized to body weight on the day of SARS-CoV-2 infection (day 56). Data are expressed as means ± standard deviation. Statistical significance between groups was calculated using an ordinary one-way ANOVA followed by Tukey’s multiple comparison test (***p<0.0002, ****p<0.0001). **(B)** SARS-CoV-2 sgmRNA levels in the nasal washes (left panel) and lungs (right panel) of MVA-S(3P)-vaccinated hamsters on day 4 post-SARS-CoV-2 infection. Data were expressed as copies per ml of nasal wash or per mg of lung tissue. Data presented as means ± SEM. The dotted line indicates the limit of detection. Statistical significance between groups was calculated using an ordinary one-way ANOVA of transformed data followed by Tukey’s multiple comparison tests (**p<0.002, ***p<0.0002, ****p<0.0001). **(C)** SARS-CoV-2 infectious particles in the lungs of MVA-S(3P)-vaccinated hamsters on day 4 post-SARS-CoV-2 infection. Data were expressed as TCID_50_ per mg lung tissue. The dotted line indicates the limit of detection. Data presented as means ± SEM. Statistical significance between groups was calculated using an ordinary one-way ANOVA of transformed data followed by Tukey’s multiple comparison test (****p<0.0001).

To assess the protective efficacy of MVA-S(3P), 4 weeks after the last dose of the MVA-S(3P) vaccine (day 56), all hamsters were infected intranasally with 2 × 10^5^ TCID_50_ of SARS-CoV-2 and then sacrificed on day 4 post-challenge (**Figure 1A**). We first assessed the change in body weight on the day of sacrifice (day 60; day 4 post-challenge) compared to the day of SARS-CoV-2 infection (day 56). This analysis shows that hamsters vaccinated with one or two doses of MVA-S(3P) had significantly increased body weight compared to animals inoculated with MVA-WT; the latter lost a mean of 5% body weight at 4 days post-challenge (**Figure 2A**).

Next, nasal washes and lung tissues obtained at 4 days post-challenge were analyzed to assess the level of SARS-CoV-2 replication. Importantly, all MVA-S(3P)-vaccinated hamsters, even after a single dose, had significant 10^2^-fold and approximately 10^5^-fold reduction (close to the limit of detection) in SARS-CoV-2 RNA levels in their nasal washes (**Figure 2B**, left) and lungs (**Figure 2C**, right), respectively, compared to the MVA-WT control group. Notably, SARS-CoV-2 infectious virus titers were undetectable in the lungs of all MVA-S(3P) vaccinated hamsters, even after a single dose (**Figure 2C**), with an approximately 10^3^-fold significant reduction, compared to the MVA-WT control group.

**Figure 3:**
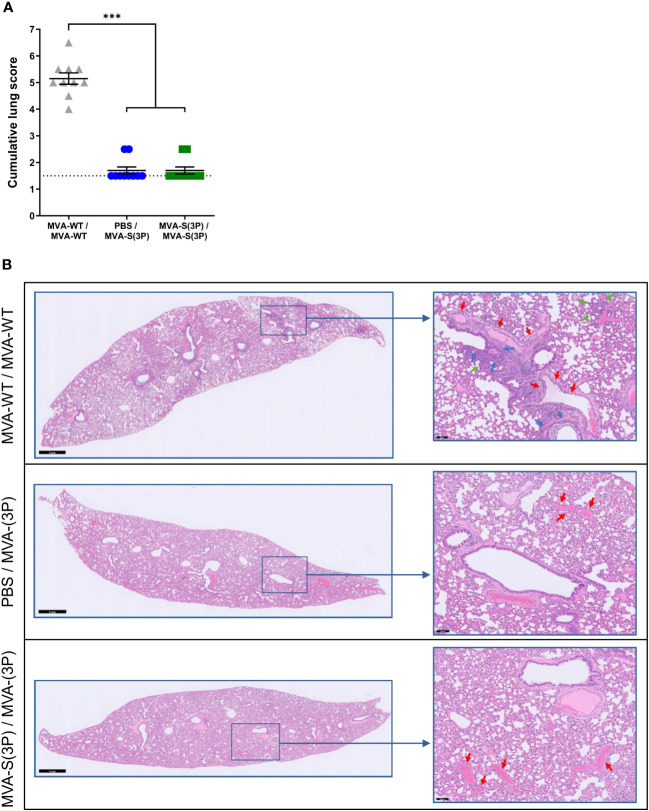
Lung histopathology. **(A)** Histopathologic scoring of hematoxylin and eosin-stained sections of hamster lungs on day 4 post-SARS-CoV-2 infection. Data presented as means ± SEM. The dotted line represents the mean lung score of uninfected hamsters (1.5). Statistical significance between groups was calculated using an unpaired non-parametric Kruskal-Wallis test (***p<0.0002). **(B)** Representative hematoxylin and eosin-stained images of lung sections from MVA-WT- and MVA-S(3P)-vaccinated hamsters on day 4 after SARS-CoV-2 infection. The general view of the lung (left) has been shown along with histopathological details of selected lung areas (blue boxes) (right). Green, red, and blue arrows (right panel) indicate bronchopneumonia, peri-vascular inflammation with endothelialitis, and peri-bronchial inflammation, respectively. Scale bars: 1 mm (left) and 100 μm (right).

Histopathological analysis of lung sections obtained 4 days post-challenge and stained with hematoxylin-eosin showed that almost no lung pathology was observed in hamsters vaccinated with one or two doses of MVA-S(3P), compared to MVA-WT control animals, with a significant reduction in cumulative lung histopathology scores that were close to the mean score of uninfected animals (**Figure 3A**). A comprehensive examination of lung sections showed that MVA-WT control hamsters had elevated scores for bronchopneumonia, perivascular and peribronchial inflammation, apoptotic bodies in the bronchial wall, and vasculitis, whereas hamsters vaccinated with one or two doses of MVA-S(3P) had residual scores close to the mean scores of uninfected animals (data not shown). Representative images of hematoxylin-eosin-stained lungs after SARS-CoV-2 infection (**Figure 3B**) showed bronchopneumonia (green arrows), significant peri-vascular inflammation with endothelialitis (red arrows), and a clear peribronchial inflammation (blue arrows) in control MVA-WT hamsters, whereas in animals vaccinated with MVA-S(3P) almost no lesions were detected and normal lung parenchyma was observed with only very focal perivascular inflammation (red arrows).

**Figure 4:**
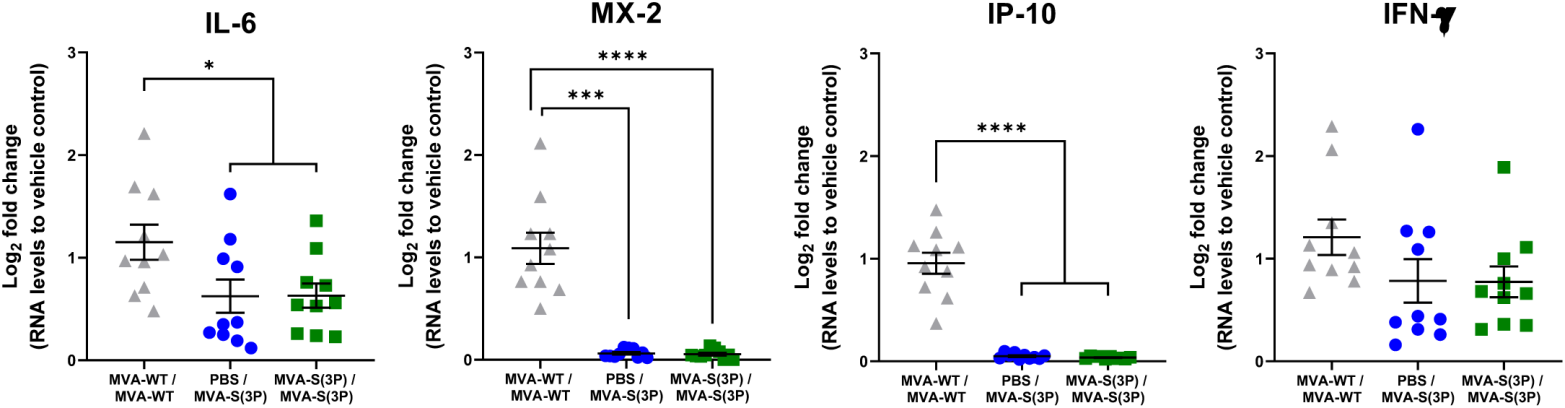
RNA Levels of proinflammatory cytokines in MVA-S(3P)-vaccinated hamsters. IL-6, MX-2, IP-10, and IFN-γ mRNA levels were detected by RT-qPCR in lungs obtained 4 days post-SARS-CoV-2 infection (n = 10/group). Mean RNA levels and SEM are depicted. The log_2_-fold change compared to the RNA levels of vehicle control is plotted. Statistical significance between groups was calculated using an ordinary one-way ANOVA of transformed data followed by Tukey’s multiple comparison test (*p<0.033, ***p < 0.0002, ****p<0.0001).

Finally, we assessed the effect of vaccination with MVA-S(3P) on the pattern of cytokine expression triggered in hamsters on day 4 post-challenge, using RT-qPCR to analyze the mRNA levels of key cytokines in lung homogenates as an index of inflammation. These data show that one and two doses of MVA-S(3P) significantly downregulated IL-6, MX-2, and IP-10 mRNA levels in a similar manner, compared to infected MVA-WT control hamsters (**Figure 4**).

## Discussion

Currently, several COVID-19 vaccines are approved and available for human use (2). Despite the good efficiency of these vaccines in reducing the severity of the disease, mortality, and hospitalizations, they still face several challenges such as their inability to prevent reinfections, limited durability of the immune responses, adverse effects related to vaccination, such as thrombosis, the emergence of new variants, complex logistics, and low availability of vaccines in low-income countries (2). Therefore, the development of novel COVID-19 vaccines is still needed to overcome the drawbacks of currently approved vaccines.

The use of MVA vectors as a vaccine platform against pathogens has been reported to elicit efficient immune responses with marked efficacy in various animal models and in human clinical trials (24, 25). Moreover, parental MVA has been approved by the European Medicines Agency (EMA) and the United States of America (USA) Food and Drug Administration (FDA) as a vaccine against smallpox and mpox. More recently, a recombinant multivalent MVA-based vaccine (MVA-BN-FILO) has been authorized by the EMA as part of a heterologous vaccination regimen for protection against Ebola virus infection (26). Regarding SARS-CoV-2, we and others have shown that MVA-based vaccine candidates against SARS-CoV-2, which mainly express SARS-CoV-2 S protein, induced robust immunogenicity and efficacy against SARS-CoV-2 in a variety of animal models, including mice, hamsters, and non-human primates (6–14, 27–40). These studies involved MVA vectors expressing either a non-stabilized form of the S protein (6–9, 12–14, 28–32, 34, 38–40) or a prefusion-stabilized S protein (10, 11, 27, 33, 35–37). Moreover, recent results of a phase I clinical trial showed that a synthetic MVA vector co-expressing SARS-CoV-2 S (in a non-stabilized version) and N proteins is safe and immunogenic, inducing SARS-CoV-2-specific humoral and T cellular immune responses (41, 42).

Recently, we reported the generation of a novel optimized non-replicating MVA vaccine candidate, termed MVA-S(3P), which expresses a prefusion-stabilized full-length S protein lacking the furin cleavage site between the S1 and S2 subunits and containing three proline amino acid substitutions in the S2 subunit, that stabilizes the S trimers in a prefusion conformation to enhance S expression in its most immunogenic form (10, 11). A single intramuscular or intranasal dose of this optimized MVA-S(3P) vaccine candidate is more immunogenic and efficacious than an MVA vector expressing a non-stabilized S protein, termed MVA-S, inducing in immunized mice higher titers of SARS-CoV-2-specific binding IgG antibodies and neutralizing antibodies, as well as Th1-type CD4^+^ and cytotoxic CD8^+^ T cell immune responses. Moreover, it enhanced the protection of transgenic K18-hACE2 mice against morbidity and mortality caused by a SARS-CoV-2 challenge (10, 11).

In the current study, we used a second animal model, that of Syrian hamsters, which is a relevant model used to assess SARS-CoV-2 pathogenesis and vaccine efficacy (13, 17, 20), to explore the immunogenicity and protective efficacy against SARS-CoV-2 infection of the MVA-S(3P) vaccine candidate. This study was performed as a necessary step to warrant the progression of this vaccine candidate to further clinical development. When compared to the MVA-WT control group, immunization of hamsters with one or two intramuscular doses of MVA-S(3P) significantly reduced SARS-CoV-2 RNA loads in the lungs and nasal washes, as well as titers of infectious virus in the lungs to undetectable levels, prevented body weight loss, and markedly reduced lung pathology. In addition, a strong reduction of IL-6, MX-2, and IP-10 RNA expression levels, genes known to be linked to COVID-19 severity (IL-6, IP-10) or at least upregulated in SARS-CoV-2 infection (MX-2) in both humans (43–45) and hamsters (17, 20, 23), was observed in the lungs of vaccinated hamsters as an indicator of protection against virus-induced lung inflammation. Remarkably, high titers of binding IgG antibodies against the S protein and neutralizing antibodies against the SARS-CoV-2 Wuhan strain and VoCs beta, gamma, and delta were detected in serum samples from vaccinated animals. Importantly, neutralizing activity against omicron BA.1 variant pseudoparticles was also detected, although variable and more dependent on repeated immunization. It remains tempting to speculate that this coverage also extends to more recent omicron subvariants (BA.4/5, BQ.1.1, and XBB) with a further evolving antigenic spectrum (46). Regarding safety, immunization of hamsters with one or two intramuscular doses of MVA-S(3P) was well tolerated with no signs of local reactogenicity or systemic vaccine-induced side effects observed in the animals.

We recently reported that the vaccine candidate MVA-S, which expresses a full-length non-stabilized S protein, is also immunogenic and effective against SARS-CoV-2 infection in Syrian hamsters (13). A direct comparison of the results obtained in Syrian hamsters immunized with MVA-S with those immunized with MVA-S(3P), obtained in this study, showed that MVA-S(3P), which expresses a full-length prefusion-stabilized S protein, is more immunogenic and effective than MVA-S. MVA-S(3P) elicited more than 10-fold higher titers of S-binding IgG antibodies and 6-25-fold higher neutralizing antibody titers against the ancestral SARS-CoV-2 Wuhan strain and several VoCs than MVA-S. Furthermore, MVA-S(3P)-vaccinated hamsters had a significant increase in body weight at 4 days post-challenge, while MVA-S-vaccinated hamsters had similar body weight compared to the beginning of the experiment. MVA-S(3P) was also more effective than MVA-S, with greater reductions in SARS-CoV-2 viral RNA (approximately 10^3^-fold) and no detectable infectious virus (40-fold reduction compared to MVA-S) in the lungs, and almost no detectable lung histopathology, whereas MVA-S-vaccinated hamsters developed small lung histopathology scores. MVA-S(3P) also led to reduced levels of several proinflammatory cytokines, while no effect was observed in MVA-S-vaccinated hamsters.

Although these results are encouraging, the hamster study has some limitations. For example, it would be interesting to determine whether MVA-S(3P) could protect hamsters against a challenge with SARS-CoV-2 omicron variants, which are now circulating among the population. In this regard, the presence of neutralizing antibodies against omicron BA.1 in hamsters vaccinated with MVA-S(3P), although at lower levels, could indicate that it is highly likely that protection against the SARS-CoV-2 omicron variant could be triggered. Future studies will examine this issue. On the other hand, in the hamster study, we did not analyze the presence of T-cell immune responses, as the reagents for evaluation of T-cell responses in hamsters are far more limited than in mice. In this regard, we previously reported that the vaccine candidate MVA-S(3P) induced robust levels of SARS-CoV-2 S-specific CD4^+^ and CD8^+^ T-cell immune responses in mice, either after intramuscular (11) or intranasal immunizations (10), so MVA-S(3P) is likely to elicit a robust magnitude of SARS-CoV-2 S-specific CD4^+^ and CD8^+^ T-cell immune responses in hamsters. Future experiments will cover this topic.

Overall, we show in this study that the MVA-S(3P) vaccine candidate is safe, highly immunogenic, and effective against SARS-CoV-2 infection in Syrian hamsters, even after administration of a single dose. These data support further clinical trial evaluation of this MVA-S(3P) vaccine candidate, whose GMP lots are already available.

## Data availability statement

The original contributions presented in the study are included in the article/supplementary material. Further inquiries can be directed to the corresponding authors.

## Ethics statement

The ethical committee of animal experimentation of KU Leuven (Belgium) approved housing conditions and experimental procedures (license P065-2020) according to institutional guidelines approved by the Federation of European Laboratory Animal Science Associations (FELASA). The study was conducted in accordance with the local legislation and institutional requirements.

## Author contributions

Conceptualization: RA, KD, ME, and JG-A. Formal analysis: RA, BW, and JG-A. Funding acquisition: RD, JN, KD, ME, and JG-A. Investigation: RA, PP, DA, GA, WK, HT, NL, and JG-A. Methodology: RA, PP, DA, GA, WK, HT, NL, and JG-A. Resources: DM, EP, and ER. Supervision: RA, LC, JN, KD, ME, and JG-A. Validation: RA and JG-A. Visualization: RA, BW, and JG-A. Writing—original draft: RA and JG-A. Writing—review and editing: all authors. All authors contributed to the article and approved the submitted version.
